# The Gender Gap in the Diagnostic-Therapeutic Journey of the Infertile Couple

**DOI:** 10.3390/ijerph18126184

**Published:** 2021-06-08

**Authors:** Giuseppe Gullo, Gaspare Cucinella, Antonio Perino, Domenico Gullo, Daniela Segreto, Antonio Simone Laganà, Giovanni Buzzaccarini, Zaira Donarelli, Angelo Marino, Adolfo Allegra, Marianna Maranto, Andrea Roberto Carosso, Piernicola Garofalo, Rossella Tomaiuolo

**Affiliations:** 1 Department of Obstetrics and Gynecology, Villa Sofia Cervello Hospital, IVF UNIT, University of Palermo, 90146 Palermo, Italy; ggullo1982@gmail.com (G.G.); gaspare.cucinella@unipa.it (G.C.); antonio.perino@unipa.it (A.P.); mariannamaranto@libero.it (M.M.); 2 Technical Panel on Gender Medicine-Sicily Regional Health Service, 90143 Palermo, Italy; mimmogullo@libero.it (D.G.); daniela.segreto@regione.sicilia.it (D.S.); piernicolagarofalo16@gmail.com (P.G.); 3 Department of Obstetrics and Gynecology, “Filippo Del Ponte” Hospital, University of Insubria, 21100 Varese, Italy; antoniosimone.lagana@uninsubria.it; 4 Department of Women’s and Children’s Health, Padova Hospital, University of Padova, 35128 Padova, Italy; 5 Psychology Unit, Andros Day Surgery Clinic-Palermo, 90144 Palermo, Italy; zairadn@libero.it; 6 Reproductive Medicine Unit, Andros Day Surgery Clinic-Palermo, 90144 Palermo, Italy; angelo.marino@clinicaandros.it (A.M.); adolfo.allegra@clinicaandros.it (A.A.); 7 Division of Gynecology and Obstetrics 1, Department of Surgical Sciences, City of Health and Science, University of Turin, 10126 Turin, Italy; andrea88.carosso@gmail.com; 8 Vita-Salute San Raffaele University, 20132 Milan, Italy; tomaiuolo.rossella@hsr.it

**Keywords:** assisted reproductive technology (ART), male infertility, gender-oriented specific approach

## Abstract

Medical procreation impairs both the biological and psychological lives of couples. However, male and female attitudes to infertility are different and require a different approach during the IVF journey. Thus, the gender impact assessment (GIA) method was used to analyse original studies present in the literature. We found some gender-related differences and, subsequently, possible outcomes of intervention to improve healthy reproduction management and prevent infertility. In particular, it became apparent that there was the need for an in-depth male infertility assessment and a gender-specific follow-up.

## 1. Introduction

Infertility affects about 15–20% of couples worldwide [1]. Despite male-factor infertility being thought to play a role in 50% of infertile couples [2], currently, most scientific studies place the male factor as a secondary consideration compared to the female factor, in contrast with other pathologies (cardiovascular, degenerative, neurological, etc.) [3]. This difference is significant when considering the psychological distress generated by infertility; there is no doubt that infertility is a stressor for a couple, but it is experienced differently by males and females [4,5].

Since procreation involves sex (biological aspects) and gender (the social construction of femininity and masculinity, which includes sociocultural and psychological aspects) [6], optimizing the diagnostic and therapeutic journey of infertility and promoting gender equality is a mandatory gender-sensitive approach.

The introduction of a sex and gender determinant in clinical practice can contribute favourably to the management of prevention, diagnosis, and treatment strategies, making health services more effective and efficient [7], as these factors influence the physiological aspect and the pathological course of diseases affecting both men and women [8].

To date, gender models affect the general behaviours of women and men, and therefore the reproductive behaviours [9,10]: although both women and men are deeply affected by the infertility diagnosis, their psychological response is significantly influenced by gender [11,12] and negatively impacts the effectiveness of diagnostic-therapeutic interventions [13,14,15]. It is necessary to analyse the relevance of gender for and within the couple and to evaluate the state of knowledge for bringing equality into the mainstream of activities [16]. Understanding the impact of gender in the development and management of reproductive health and infertility can benefit the couple in terms of intervention and outcome and provide a deeper understanding for researchers and clinicians [17].

This paper aims to assess the impact of the gender dimension on the diagnostic-therapeutic journey of infertile couples and identify some crucial intervention points in order to address the gender balance. In particular, we adopted the gender impact assessment (GIA) method, validated for law and social issues, and applied it to a public health system [18].

## 2. Materials and Methods

A nonsystematic review was done through a search on the following databases: MEDLINE, EMBASE, Global Health, The Cochrane Library (Cochrane Database of Systematic Reviews, Cochrane Central Register of Controlled Trials, Cochrane Methodology Register), Health Technology Assessment Database, Web of Science, and research registers (such as www.cliniclatrials.gov (accessed on 1 March 2021); we used the medical subject heading (MeSH) terms “Gender Identity” (MeSH Unique ID: D005783) or “Gender Role” (MeSH Unique ID: D000085402) in combination with “Infertility” (MeSH Unique ID: D007246), “Infertility, Male” (MeSH Unique ID: D007248), “Infertility, Female” (MeSH Unique ID: D007247), and “Reproductive Techniques, Assisted” (MeSH Unique ID: D027724). We selected papers written in English, with no time restrictions regarding the year of publication.

Titles and/or abstracts of studies retrieved using the search strategy, and those from additional sources, were screened independently by two review authors (A.S.L. and R.T.) to identify studies that potentially meet the aims of this nonsystematic review. These potentially eligible articles’ full text was retrieved and independently assessed for eligibility by the other two review team members (G.G. and G.B.). Any disagreement between them over the eligibility of particular articles was resolved through discussion with a third (external) collaborator. Two authors (A.R.C. and P.G.) independently extracted data from articles about study features and included populations, type of intervention (duration of therapy and drug posology), and outcomes. Any discrepancies were identified and resolved through discussion (with a third external collaborator where necessary). Due to the nature of the findings, we opted for a narrative synthesis of the selected articles’ results.

To detect disparities and degrees of difference in the diagnostic-therapeutic journey of infertile couples, the gender impact assessment (GIA) method was chosen [18]. This method is a stepwise approach which, through the identification of relevant gender issues, picks up gendered effects and simulates gender equality outcomes. The basis for identifying gender relevance is to disaggregate the data by sex, subsequently carry out the full-fledged gender impact assessment, and finally address the gender balance with suggestions for reducing gender inequalities and promoting gender equality. In particular, we performed the analysis through the following macrosteps.

1. Pretest of checking gender relevance.
2. Full-fledged gender impact assessment (GIA), identifying and evaluating gender impacts.
3. Addressing the gender balance, giving suggestions for reducing gender inequalities and improving gender equality.

Every article included in the review was carefully read, and qualitative data were identified and extracted. Key themes have been identified and used to create a narrative discussion due to the difficulty in obtaining a quantifiable parameter. The parameters considered as relevant were the following:

1. Characteristics of the subjects: age/date of birth, nationality, educational qualification, profession, religion, and relationship with ART.
2. Characteristics of the families of origin: age of parents; profession; living distances; welfare needs; years of marriage and procreative research; sequential reconstruction of the family story accompanied by age, marital status, and presence of children; the possible presence of cases of abortion or sterility in the family; and any genetically transmitted diseases or infections.
3. Story of the couple: years of engagement, marriage/cohabitation, and coital frequency; any previous relationships; significant experiences faced together; the story of the personal process of procreative waiting and health; causes of infertility and any surgical intervention (e.g., varicocele, endometriosis, etc.); and previous ART.
4. Psychological interview: biopsychosocial data collection, its usefulness, and other contacts with psychologists in the past.

## 3. Discussion

Gender bias in health involves biological sex differences and gender differences in the way women and men behave and how they are treated [17]. Gender bias is challenging to eliminate. However, we argue that the gender impact assessment can become a force for good in moving health practices towards gender equity by revealing and challenging gender bias. The gender impact assessment (GIA) can be defined as an ex ante or ex post evaluation, analysis, or assessment of law, policy, or programme that helps to identify the likelihood of a decision having negative consequences for equality between women and men [18]. GIA is aimed at improving the design and planning policy to prevent a negative effect on gender equality and improve gender equality through gender-oriented strategies [19].

**Figure 1:**
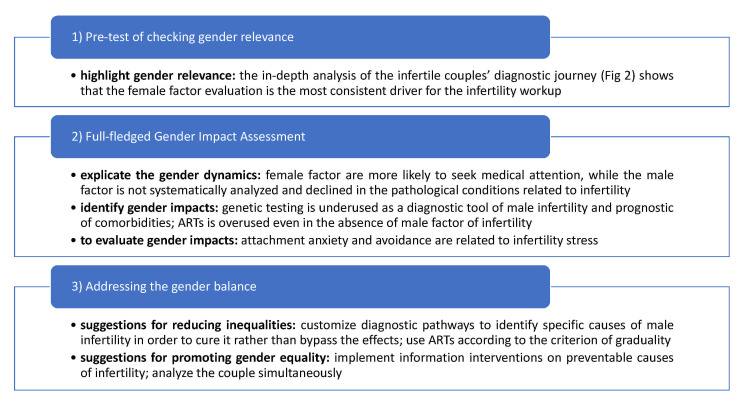
The steps of GIA applied to couple infertility. To obtain disaggregate data by sex, the diagnostic-therapeutic journey of infertility was analysed separately for males and females. Genetic tests, assisted reproduction techniques, and psychological factors were chosen as indicators to assess the gender impacts.

To date, the GIA tool has been recently assessed for health practice [20]. In particular, a large cohort of healthcare employees participating in the Italian vaccination campaign against SARS-CoV-2 has been investigated to assess the impact of sex and gender on vaccination coverage using the GIA approach. However, couple infertility is perfect for GIA analysis due to the implication of both sex and gender factors. Since infertility always harms both partners, it can be the first healthcare issue in which a systematic gender impact assessment could be efficiently performed and subsequently used in other health settings. The step-by-step method and the paired results are summarized in Figure 1.

**Figure 2:**
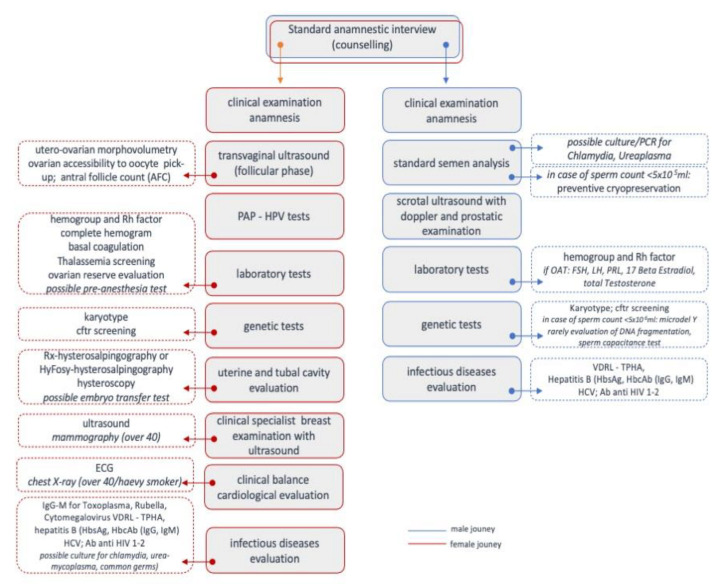
The infertile couple is subjected to instrumental and laboratory investigations to define the most appropriate diagnosis and therapy. The figure shows the most common diagnostic examinations prescribed to the women (red line) and the men (blue line), as derived by the analysis of the studies and the experience of the ART clinics involved in this study.

The first step of GIA applied to the journey of couple infertility showed that gender is a relevant determinant. Although infertility affects both men and women equally, the in-depth analysis of the infertile couples’ diagnostic journey (Figure 2) shows that the female factor evaluation is the most consistent driver for the infertility workup.

The full-fledged gender impact assessment (second step) focused on explaining the gender dynamics and identifying and evaluating gender impacts. Owing to social constructs, current gender dynamics mean that female factors are more likely to seek medical attention. In contrast, male factors are not systematically analysed and overlooked in the specific pathological conditions related to infertility [21].

The harmful impacts of gender bias were investigated through genetic testing and ART. Currently, male infertility’s genetic diagnostic workup, restricted to the karyotype, AZF region, and CFTR gene, is considered ineffective [22]. About 2000 genes are known to be involved in spermatogenesis. The number of genetic variants related to infertility is constantly increasing, providing a decrease in the current percentage of idiopathic male infertility [22]. Furthermore, numerous data suggest that genetic abnormalities involved in male infertility can also affect morbidity and life expectancy, suggesting a link between male infertility and oncological, cardiovascular, metabolic, and autoimmune diseases [2].

The ART, which has rapidly evolved during the last decades, led to overcoming the limitations due to male factor infertility [23], and in particular, the development of intracytoplasmic sperm injection (ICSI) can currently be considered a significant step forward [24]. Furthermore, ICSI has no clear advantages in patients with normal semen parameters and should be offered only in cases of severe male factor [25]. Indeed, ICSI is not associated with a significantly higher fertilization rate, clinical pregnancy rate, implantation rate, and live birth rate than standard IVF in couples without male factor infertility. These findings have been confirmed even in the presence of more mature women and, consequently, worse oocyte quality [26,27,28]. Only recently, growing evidence suggests that sperm carries pivotal factors acquired intrinsically during spermatogenesis or extrinsically during storage and ejaculation in the male reproductive tract. Indeed, spermatozoa’s role has been neglected for too long, consequent to systematic use of ICSI [29].

On this basis, in a future scenario, the male partner should be carefully managed by taking into account not only the sperm concentration, motility, and morphology, but also genetic tests and testicular histology in order to offer a tailored treatment and targeted use of ART [30].

Finally, psychological aspects were considered for the assessment of gender impacts. Many couples state that infertility is the most stressful problem in their lives and that fertility treatments disrupt individuals’ and couples’ lives [6,31,32,33,34]. Specifically, the large body of literature focused on different variables (behavioural, relational, social, emotional, and cognitive) across treatment stages (before, during, and after treatment) involved in infertility highlighted the need for implementing routine care to minimize the impact of psychosocial infertility threats [31]. The emotional (depression, anxiety, stress/distress, psychopathology, psychiatric disorders, general well-being, quality of life, etc.) and relational/social issues (relationship with partner, family, friends and more extensive social network, and work) have primarily been studied by taking into account the female partner. Only the most recent literature suggests that clinical work and research should involve male partners and focus on the couple level [31]. It has been recognized that men’s anxiety symptoms may develop primarily during their fertility treatment [35]. Self-reported sleep disturbances, sleep duration, and late bedtimes have all been associated with poor semen quality [36], even if evidence of sleep issues is reported during the early stages of fertility treatment, in only one study [37]. Moreover, several studies show that psychological stress among men during infertility treatment is not associated with testicular function [38]. Men often experience low self-esteem and lack of adequacy in their social and familial role and appear to experience a lower emotional impact on fertility problems than women [39,40,41,42]. Still, several mechanisms could lead studies to under-report levels of infertility-related distress among men. First of all, men tend to suppress their emotions to support their partners [43,44]. Their commonly used active-avoidance and passive-avoidance coping [45] and their attachment anxiety and avoidance are associated with infertility stress [46,47]. The European Society of Human Reproduction and Embryology (ESHRE) guidelines indicate that being a man, having a lower educational level, and having treatment-related physical or emotional complaints are risks for experiencing increased relational and social problems during a treatment period [31]. Secondly, it has also been highlighted that men report higher social isolation than women and are not likely to refer to psychosocial services [48].

What can be learned from many of the studies focusing on cognitive, emotional, behavioural, relational, and social issues highlights both men and women as well as the relevance of offering specific psychological counselling to both because they are strongly connected to each other. Individuals who perceive their partner to be available and responsive experience lower infertility stress than individuals who perceive their partner as avoidant and nonresponsive [46,47]. In couples, the way one partner reacts to the infertility condition/diagnosis [48] and its treatment [46] is associated with how the other partner reacts [47,49,50]. Men’s attachment anxiety is related to their partners’ infertility stress [47]. A partner’s use of active-avoidance coping is related to higher marital distress for men and women [51]; a partner’s use of meaning-based coping is associated with lower marital distress in men, reinforcing the dyadic nature of data when approaching infertility issues. Based on these data, psychological interventions detecting and treating individual and dyadic psychological needs of couples involved in fertility journeys are strongly recommended. Finally, counsellors should focus their intervention on helping infertile people consider treatments as one of the potential opportunities to reshape their global aims and adopt a broader perspective of their entire lives.

Taking all these data together, we can summarize that the GIA of the diagnostic-therapeutic journey of the infertile couple has brought out some key points. All data are grouped together in Table 1, where the state of the art according to our review is presented, and the strategies to reduce the gender differences impact on ART are proposed.

First, there is a strong tendency towards medicalization rather than preserving healthy reproduction. ART represents the apical moment of the reproductive medicalization process, bypassing the couple’s infertility rather than resolving the causes. ART is increasingly used, neglecting that in many cases, infertility can be prevented with effective awareness campaigns (age, sexually transmitted diseases, lifestyle, and environmental pollution) or resolved with in-depth targeted therapeutic, diagnostic interventions [52]. Where the indications allow it, the therapeutic choices should follow the principle of graduality and should be differentiated into subsequent steps [22].

Secondly, the diagnostic-therapeutic phase involves the female partner more, to the detriment of the male counterpart. Due to the concept that the infertile couple must be considered as a single entity [22], the diagnostic process should be carried out on both partners simultaneously [52] to improve a couple’s reproductive choices. Therefore, both partners must undergo thorough investigations to rule out potentially reversible causes of infertility and improve the ability to procreate naturally. Furthermore, the negative impact of an unbalanced diagnostic process is reflected in the lack of diagnosis for the underlying causes of male infertility. Improving the detection rate of genetic tests also becomes essential because male infertility is associated with poorer overall health, increased cancer risk, and decreased life expectancy. Therefore, general practitioners have a pivotal role in educating patients about modifiable factors, maximizing the fertility potential, and improving the male patient’s overall health [53].

The third consideration that emerged is the male partner’s psychological distress, mainly due to the different involvement in the whole diagnostic-therapeutic process [8]. Infertility should be addressed using a gender-specific-related approach. Indeed, the gender-specific approach takes into account the recent criteria of intersectionality [54]: it highlights how various biological, social, and cultural categorizations (for example, gender, ethnicity, social class, disability, sexual orientation, religion, age, nationality, species, and other interconnected axes of identity) interact at multiple levels, often simultaneously, being able to create factors of discrimination and inequalities [55,56]. Through an anamnesis conducted with the awareness of intersectionality, the differences and traits of a person are recognized as inextricably linked to all the other elements, with the advantage of being able to understand better that person’s identity and more or less manifest needs and being able to both cure it (caring) and take care of it (curing) in a “tailored” and “gender-specific” way [57,58,59].

A fourth consideration can be proposed from the authors’ perspective according to the need to address the gender perspective in ART in a wider vision. First, it must be considered that ART is prone to gender differences which are continuously studied and intrinsic in the treatment itself. For example, it has been shown that significantly more males are born after blastocyst transfer in IVF cycles [60]. Although further studies are needed to detect real differences and aetiology, this issue makes the gender impact debate intriguing. Secondly, in the last 10 years a new movement has grown called low-cost IVF (LCIVF) activism. This movement’s aim relies on making IVF more accessible for women in every country of the world. This effort is made by ESHRE and finds its main key points in milder stimulation protocols, single-embryo transfers, and cost-effectiveness of infertility diagnosis and treatment [61]. Since LCIVF may become the future of ART in developing countries, a strong effort should, in our opinion, be made ex ante in assessing gender impact and preventing gender differences.

## 4. Conclusions

**Figure 3:**
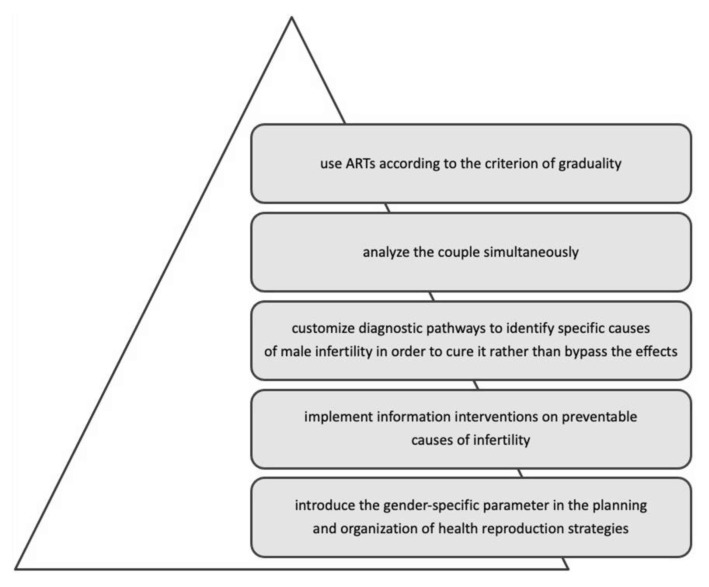
Crucial points of intervention for improving couple infertility management with a gender-oriented approach.

The integration of scientific knowledge with gender-specific determinants has brought out some crucial points of intervention (Figure 3): the need to implement information interventions on preventable causes of infertility; customize diagnostic pathways to identify specific causes of male infertility to cure it rather than bypass its effects; analyse the couple simultaneously; and use ART according to the criterion of graduality.

In conclusion, including the gender dimension throughout the diagnostic-therapeutic journey of infertile couples helps eliminate gender bias, indicating how to design equitable access to infertility diagnosis and treatment.

## Figures and Tables

**Table 1:** The problems affecting gender impact in ART are presented as “as is”. Strategies to reduce gender differences impact are presented as “to be”. Results are strictly linked to gynaecological and genetic assessment strategies in ART.

AS IS (Problems)	TO BE (Solutions)
The erroneous concept that full investigation for infertile men is not needed	Male fertility experts should always be involved in the diagnostic process of the infertile couple
Male infertility is usually defined only based on semen analysis	Assessment should embrace: Semen microbiological examination; Endocrine assessment; Genetic tests, also using next-generation sequencing (NGS); Testicular histology.
Semen reporting is still performed in many laboratories that do not have adequate preparation	Semen should be evaluated according to the World Health Organization (WHO) manual and preferably performed in laboratories that have expertise in reproductive medicine
A common malpractice is: To look only at sperm concentration, motility, and morphology; Not performing double semen analysis before making a diagnosis.	Solutions are: Consider total sperm count per ejaculate, rather than sperm concentration per millilitre, because it better reflects testicular and seminal tract function; Consider that the interval between the two semen analyses should ideally be 2–3 months when acute illnesses or medical treatment interfering with spermatogenesis occurred.
Multiple cycles of IVF/ICSI can last for years and the male figure must not be neglected during the months of treatment, limiting itself to the sole observation of the seminal fluid values	In addition, given the strong association between infertility, cryptorchidism, testicular hypotrophy, and microlithiasis with testicular cancer, recurring scrotal ultrasonography is a great opportunity to identify suspected testis masses and nodules
Genetic variables are studied: Through a direct search for mutations; With fragmented investigations; With high costs.	Solutions are: Using a genomic scanning method; NGS-based method reduces costs and time.

## Data Availability

All data are provided within this study.
